# Antibody response against selected epitopes in the HIV-1 envelope gp41 ectodomain contributes to reduce viral burden in HIV-1 infected patients

**DOI:** 10.1038/s41598-021-88274-9

**Published:** 2021-04-26

**Authors:** Rute Marcelino, Filipa Gramacho, Francisco Martin, Pedro Brogueira, Nuno Janeiro, Claudia Afonso, Robert Badura, Emília Valadas, Kamal Mansinho, Luís Caldeira, Nuno Taveira, José M. Marcelino

**Affiliations:** 1 Global Health and Tropical Medicine-GHTM, Instituto de Higiene e Medicina Tropical-IHMT, Universidade Nova de Lisboa-UNL, 1349-008 Lisboa, Portugal; 2 Hospital de Santa Maria-HSM, Centro Hospitalar Lisboa Norte-CHLN, E.P.E., Lisboa, 1649-028 Lisboa, Portugal; 3 Clínica Universitária de Doenças Infeciosas, Faculdade de Medicina, Universidade de Lisboa-UL, Lisboa, 1649-028 Lisboa, Portugal; 4 Serviço de Doenças Infeciosas, Hospital Egas Moniz-HEM, Centro Hospitalar Lisboa Ocidental-CHLO, E.P.E., Lisboa, 1349-019 Lisboa, Portugal; 5 Instituto de Investigação do Medicamento (iMed.ULisboa), Faculdade de Farmácia, Universidade de Lisboa, Lisboa, 1649-003 Lisboa, Portugal; 6 Centro de Investigação Interdisciplinar Egas Moniz (CiiEM), Instituto Universitário Egas Moniz, Monte de Caparica, 2829-511 Monte de Caparica, Portugal

**Keywords:** Vaccines, Virology

## Abstract

The ectodomain of gp41 is the target of potent binding and neutralizing antibodies (NAbs) and is being explored in new strategies for antibody-based HIV vaccines. Previous studies have suggested that the W164A-3S (3S) and EC26-2A4 (EC26) peptides located in the gp41 ectodomain may be potential HIV vaccine candidates. We assessed 3S- and EC26-specific binding antibody responses and related neutralizing activity in a large panel of chronic HIV-1-infected Portuguese individuals on ART. A similar proportion of participants had antibodies binding to 3S (9.6%) and EC26 (9.9%) peptides but the level of reactivity against 3S was significantly higher compared to EC26, except in the rare patients with double peptide reactivity. The higher antigenicity of 3S was unrelated with disease stage, as assessed by CD4^+^ T cell counts, but it was directly related with plasma viral load. Most patients that were tested (89.9%, N = 268) showed tier 1 neutralizing activity, the potency being inversely associated with plasma viral load. In the subset of patients that were tested for neutralization of tier 2 isolates, neutralization breadth was inversely correlated with plasma viral load and directly correlated with CD4^+^ T cell counts. These results are consistent with a role for neutralizing antibodies in controlling viral replication and preventing the decline of CD4^+^ T lymphocytes. Importantly, in patients with 3S-specific antibodies, neutralizing titers were inversely correlated with viral RNA levels and proviral DNA levels. Moreover, patients with 3S and/or EC26-specific antibodies showed a 1.9-fold higher tier 2 neutralization score than patients without antibodies suggesting that 3S and/or EC26-specific antibodies contribute to neutralization breadth and potency in HIV-1 infected patients. Overall, these results suggest that antibodies targeting the S3 and EC26 epitopes may contribute to reduce viral burden and provide further support for the inclusion of 3S and EC26 epitopes in HIV-1 vaccine candidates.

## Introduction

Despite recent progress in reducing the number of new infections, 1.7 million [1.2 million–2.2 million] people became newly infected with HIV in 2019 and HIV/AIDS is still among the leading causes of disease burden and mortality in sub-Saharan Africa^1^. Current projections indicate that most countries are not on track to meet the Sustainable Development Goal (SDG) 3 of “Ending the AIDS pandemic as a public health threat by 2030”^2^.

Eliminating HIV from the human population will require a successful vaccine. However, candidate vaccines evaluated to date have either failed or have shown very modest efficacy. A successful HIV-1 vaccine should elicit potent and broadly neutralizing antibodies similar to those found in some HIV-1 controllers^3–5^. Such antibodies may protect patients from disease progression and can neutralize a wide range of genetically diverse HIV-1 subtypes. Many different broadly neutralizing monoclonal antibodies (bnAbs) targeting epitopes in gp120 or gp41 have been isolated from HIV controllers and elite neutralizers^5^. These bnAbs allowed the identification of key vulnerable sites on the HIV-1 envelope glycoprotein (gp120/gp41) and are guiding the design of the new generation of HIV vaccines^6^. More than 100 clinical trials have been performed in last 30 years to evaluate HIV vaccine candidates. So far, no vaccine has succeeded in inducing bnAbs, as noted in the Thai Phase III clinical trial (RV144) conducted in Thailand in 2009 and in the clinical trial (HVTN 702) that stopped in April 2020^4, 5, 7^.

The huge diversity of HIV surface envelope glycoprotein (gp120) is one of the obstacles to the development of an antibody-based vaccine^8, 9^. Therefore, some efforts have been concentrated on highly conserved regions of the gp41 ectodomain, including the fusion peptide, the heptad repeat 2 (HR2) and the membrane proximal external-region (MPER)^6, 10, 11^. These regions play key roles in membrane fusion. MPER is the target of at least seven potent bnAbs named 2F5, 4E10, 10E8, DF51, Z13e1, VRC42 and CH12, which inhibit between 80 and 100% of the HIV-1 primary isolates tested^5, 6, 12–14^. However, despite numerous attempts using different strategies, bnAbs against MPER have been difficult to elicit in animal models and the few vaccines developed based on MPER epitopes elicited only neutralizing antibodies of low potency and limited breadth^5, 13–15^.

The 28-mer EC26-2A4 peptide (EC26 for short) (aa 646–673, in HIV-1 HXB2) is located in the HR2 region of the gp41 ectodomain and contains the NH2-half of the MPER including the epitope of the 2F5 broadly neutralizing antibody (662- ELDKWAS- 668)^6, 16–18^. The neutralizing epitope of this peptide overlaps that of 2F5 (aa 659-ELLELKDWA-667). In mice, EC26 peptide conjugates and a optimized version of the peptide named EC26-2A4-ΔM elicited the prodution of antibodies that neutralized tier 1 HIV-1 strains and in the case of EC26-2A4ΔM did not react with cardiolipin and phospholipids^19–21^.

The 15-mer 3S peptide (aa 609–623) is located between heptad repeat 1 (HR1) and HR2, and contains the epitope 613-SWSNKS-618^21–24^. A single W-to-A substitution at position 614 originated peptide W164A-3S; anti-W164A-3S antibodies with neutralizing activity and conferring protection from CD4^+^ T cells depletion were detected in the sera of some HIV-1 patients^25^. In a cohort of long-term non progressors neutralizing antibodies targeting W164A-3S were correlated with low viral and proviral loads, and were associated with preservation of high CD4^+^ T-cell counts and T-cell responses^26^. The immunogenicity of W164A-3S was tested in mice eliciting the production of broadly neutralizing antibodies^25^. More recently, in a therapeutic HIV-1 vaccine phase I / IIa clinical trial, W164A-3S increased the number of CD4^+^ T cells with a non-exhausted phenotype^24^.

Due to their neutralizing potential and the capacity of anti-W164A-3S antibodies to reduce CD4^+^ T cells depletion, EC26 and W164A-3S are considered good vaccine candidates^21, 27^. Since HIV-1 is a highly variable virus and this may affect in particular the performance of epitope based vaccines^28^, a global survey of the antigenicity of W164A-3S and EC26 is required to better inform vaccine design and production efforts. In this study we characterized the antigenicity of W164A-3S and EC26 epitopes in a large panel of chronically infected HIV-1-infected individuals from Portugal on ART and the impact of W164A-3S and EC26 antibodies in disease stage and viral burden. Our results suggest that antibodies targeting the W164A-3S and EC26 epitopes may contribute to reduce viral burden and provide further support for the inclusion of W164A-3S and EC26 epitopes in HIV-1 vaccine candidates.

## Results

### Characteristics of the patients

The characteristics of the patients enrolled in this study are shown in Table 1. All patients were on antiretroviral therapy (ART) for more than 6 months and most were aviraemic (622, 89.5%). Most individuals were male (497, 71.5%). The median CD4^+^ T cells, nadir CD4^+^ T cell counts and CD4/CD8 ratio were significantly lower in viraemic than in aviraemic patients. The age and time since HIV diagnosis were also significantly lower in viraemic patients. In contrast, proviral load levels in PBMCs were similar in both groups which may be due to the low number of viraemic patients.

**Table 1:** Characteristics of HIV-1 infected patients enrolled in this study.

Parameters, median (IQR)	Aviraemic patients	Viraemic patients	*P* value
	(N = 622)	(N = 73)	
**Gender (%)**			
Male	451 (72.5)	46 (63.01)	0.1001
Female	171 (27.5)	27 (36.99)	
Age, years	48 (42–56)	42.5 (36.3–49.0)	< 0.0001
Time since HIV-1 diagnosis, years	14.4 (9.2–18.4)	11.5 (6.8–17.4)	0.0049
CD4^+^ T cells (cells/mm^3^)	669 (497–890)	304 (152–519)	< 0.0001
Nadir CD4^+^ T cells (cells/mm^3^)	200 (78–329)	127 (44.5–241)	0.0016
CD8^+^ T cells (cells/mm^3^)	852 (618–1179)	1148 (767–1536)	< 0.0001
CD4^+^/CD8^+^ ratio	0.798 (0.526–1.116)	0.268 (0.130–0.409)	< 0.0001
HIV-1 RNA (log_10_ copies/mL)	LDL	2.61 (2.13–4.01)	na
HIV-1 DNA (log_10_ copies/10^6^ cells)	2.49 (2.02–3.06)	2.79 (2.23–3.18)	0.1694
	(N = 233)	(N = 23)	

IQR, interquartile range; LDL, lower than the detection limit of 20 copies of HIV-1 RNA/mL plasma; na, not applicable; Statistical significance (*P* value) was determined by using the Mann–Whitney test or Fisher’s exact test.

### Antibody binding reactivity against gp41 peptides

**Figure 1:**
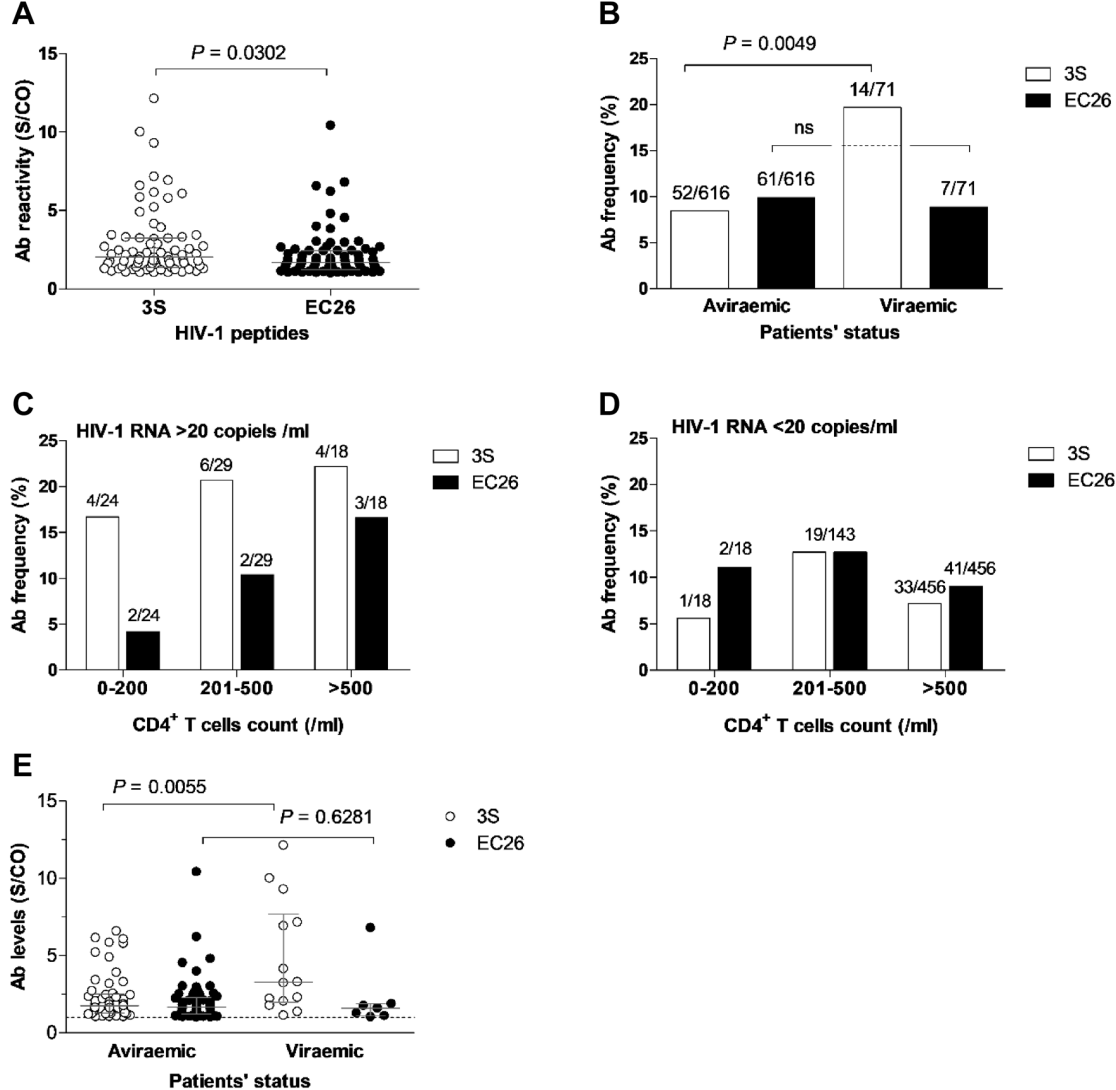
3S and EC26-specific antibody frequency and reactivity in plasma from HIV-1-infected Portuguese. (**A**) Peptide-specific antibody reactivity levels in all patients; (**B**) Proportion of HIV-1 plasma samples reacting with the peptides in viraemic and aviraemic patients; **C** and **D**) Antibody frequency according to HIV RNA and CD4^+^ T cells counts; (**E**) Plasma reactivity levels against both peptides in viraemic and aviraemic patients. Lines denote the median and interquartile range. S/CO (OD sample/OD cut-off ratio), values ≥ 1 (dotted line) indicate reactive samples. P values refer to the comparison between groups and were determined using the Mann Whitney test. In all figures, white symbols represent 3S-specific responses and black symbols EC26-specific responses.

The percentage of samples reacting with 3S or EC26 peptides was similar (N = 66, 9.5% and N = 68, 9.8%, respectively) but the level of reactivity against 3S was significantly higher compared to EC26 [median binding (IQR) = 1.87 (1.34–3.30) vs. 1.64 (1.21–2.20), *P* = 0.0302] (Fig. 1A). Moreover, reactivity to 3S but not to EC26 was directly related to viral replication. Thus, a similar frequency of viraemic and aviraemic patients (9.90% vs 9.86%) reacted with EC26, but much higher frequency of viraemic patients reacted with 3S relative to aviraemic patients (19.72% vs. 8.44%, *P* value = 0.0049) and this was irrespective of the number of CD4^+^ T cells (Fig. 1B–D). Likewise, the antibody reactivity level against 3S was significantly higher in viraemic patients compared to aviraemic patients [median binding (IQR) = 3.27 (1.98–7.68) vs. 1.76 (1.28–2.52), *P* = 0.0055], while no significant difference was observed for EC26 [median (IQR) binding = 1.59 (1.11–1.88) vs. 1.66 (1.21–2.29), *P* = 0.6281] (Fig. 1E).

**Figure 2:**
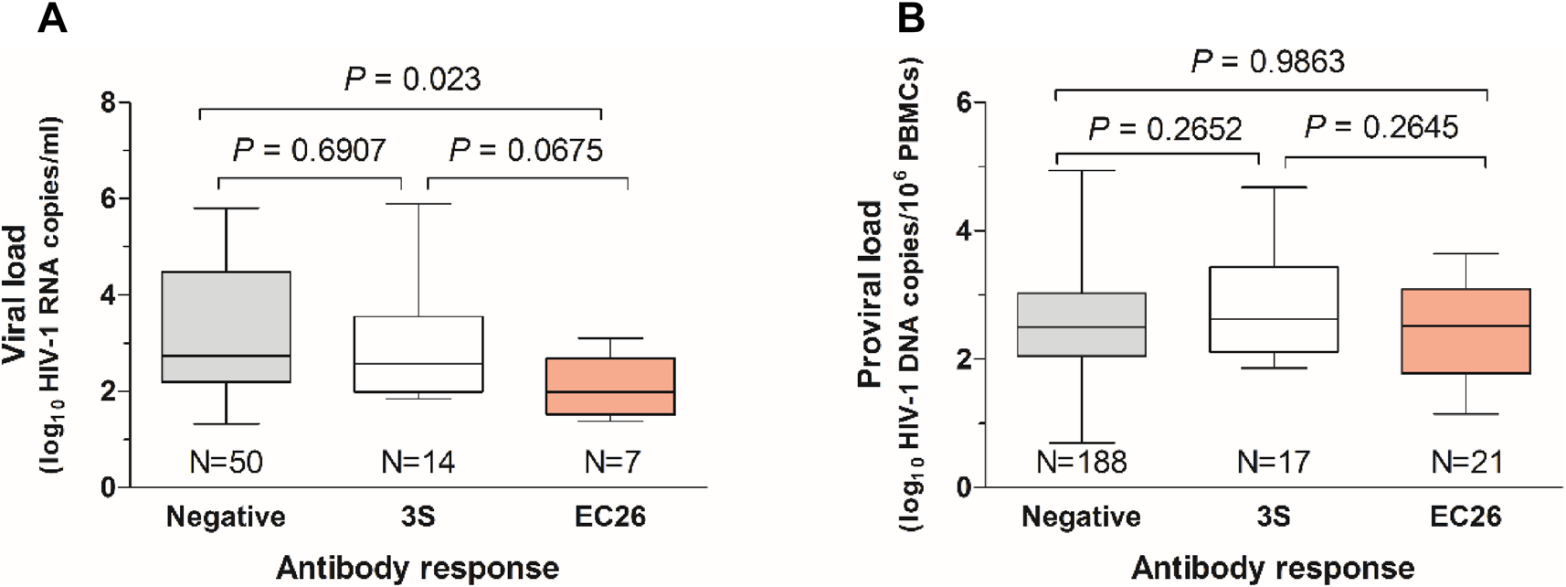
Box and whiskers plot showing the relationship between reactivity to 3S and EC26 and viral load (**A**) and proviral load (**B**). Lines denote median, maximum and minimum values. Median values were compared using the Mann–Whitney test.

Interestingly, in viraemic patients, viral load was lower in patients with antibodies against EC26 relative to patients with no antibody reactivity or with antibodies against 3S. This difference reached statistical significance when comparing patients with no antibodies with patients with EC26 antibodies (2.74 vs. 1.99 median log_10_ HIV-1 RNA copies/mL; *P* = 0.023) (Fig. 2A). Reactivity against both peptides was unrelated with proviral DNA levels (Fig. 2B).

Overall, these results show that 3S is more antigenic than EC26 in this population and this is directly related with viral load in plasma. On the other hand, the lower viral load in patients with EC26-specific antibodies suggests a potential role for these antibodies in controlling viral replication.

**Figure 3:**
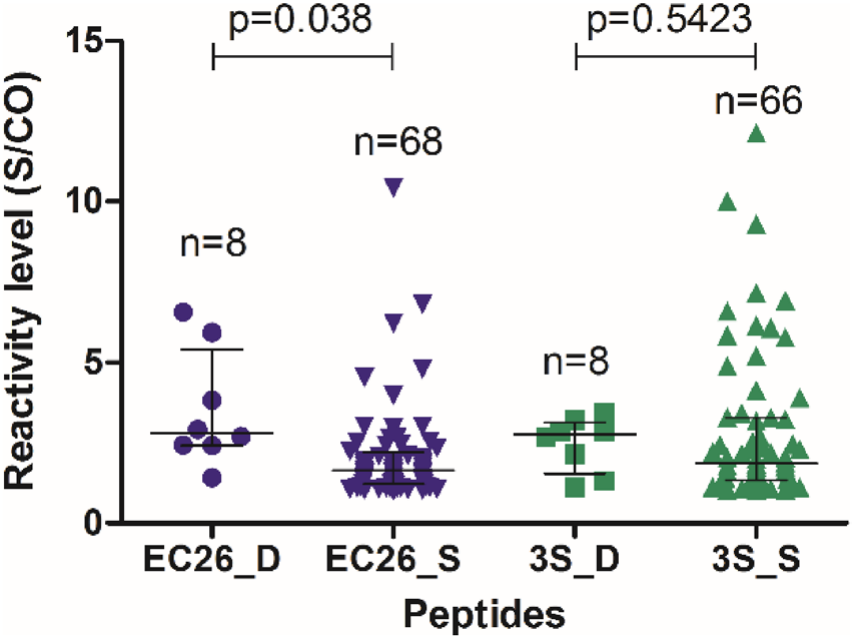
Level of reactivity against the 3S and EC26 peptides in plasma samples from patients showing single (S) or double (D) reactivity against 3S and EC26. Lines denote median and interquartile range. Statistical significance was determined using the Mann–Whitney test.

Only eight (1.2%) patients had antibodies against the two peptides (Table S1). Interestingly, in these patients the level of reactivity against EC26 and 3S was higher than in patients with single reactivity (1.7-fold for EC26 and 1.5-fold for 3S) (Fig. 3). Moreover, in contrast to patients with single reactivity, in these patients the EC26 reactivity exceeded that of 3S. The rarity of simultaneous antibody responses against both peptides indicates that the simultaneous exposure of the 3S and EC26 antigenic epitopes in gp41 is uncommon during infection.

### Neutralizing antibody response

**Figure 4:**
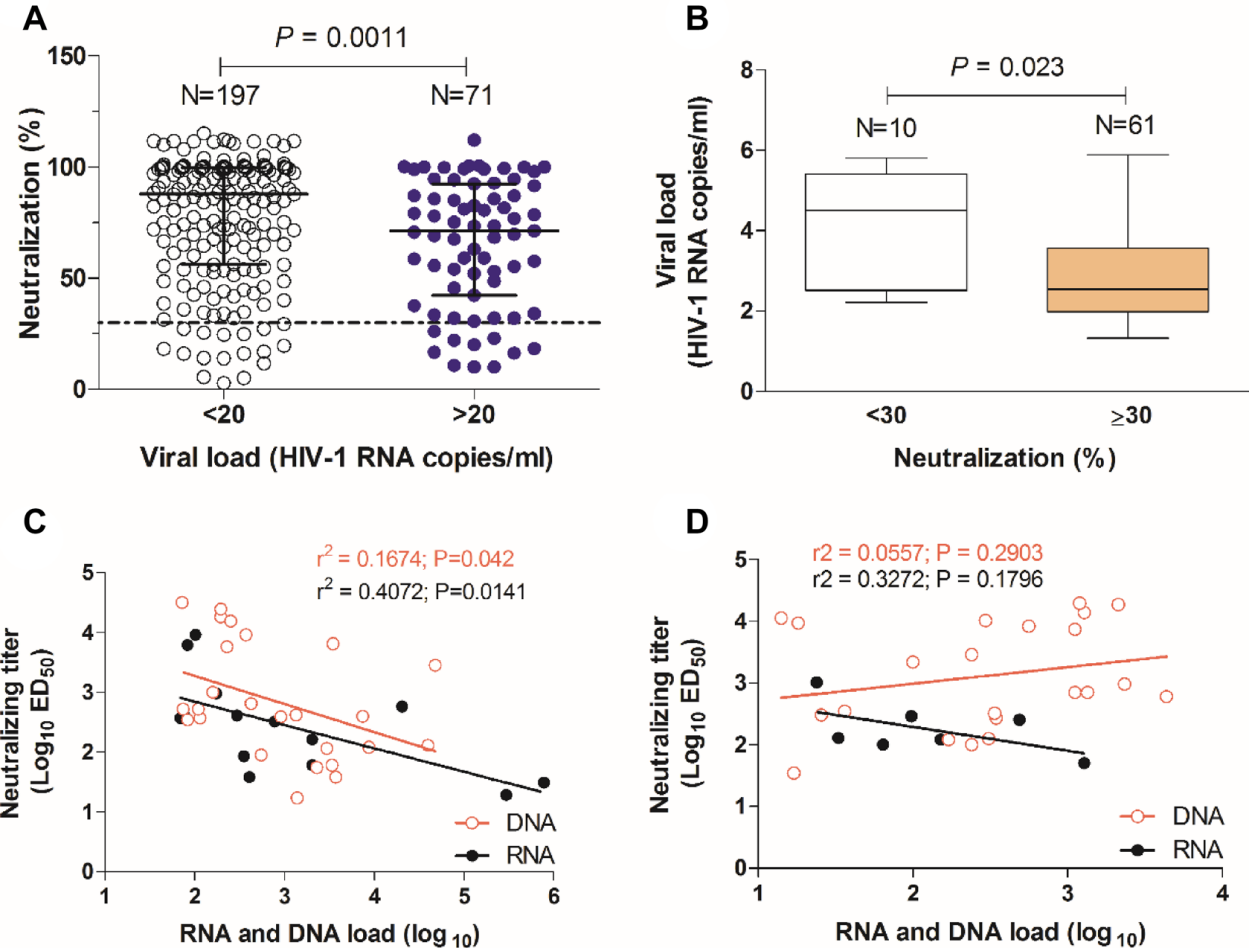
Neutralizing antibody response in relation with viral burden and the presence of 3S or EC26-binding antibodies. (**A**) Percent neutralization of HIV-1 strain NL4-3 (Tier 1a virus) using plasmas from patients with detectable and undetectable viral load. Lines denote the median and interquartile range; (**B**) Box and whiskers plot of viral load in patients with and without neutralizing antibodies. Lines denote median, maximum and minimum values; (**C**) Correlation of neutralizing titers with RNA and DNA load in patients containing 3S-specific antibodies (N = 31); (**D**) Correlation of neutralizing titers with RNA and DNA load in patients containing EC26-specific antibodies (N = 28). The linear regression lines, goodness of fit r^2^ and *P* values are indicated.

Neutralizing antibodies (nAbs) in plasmas were first assessed against subtype B HIV-1 NL4-3 isolate, a tier 1A isolate (easy to neutralize). Of the 268 plasma samples that were tested (197 from aviraemic patients and 71 from viraemic patients), 241 (89.9%) showed neutralizing activity ≥ 30% and the neutralizing potency was higher in aviraemic patients than in viraemic patients (median 87.8% neutralization vs. 71.3%, *P* = 0.0011, respectively) (Fig. 4A). In viraemic patients, the median viral load was 1.8-fold lower in patients with neutralizing activity ≥ 30% compared to those without neutralizing activity (< 30%) (median log_10_ HIV RNA copies/mL 2.5 vs. 4.5, *P* = 0.023) (Fig. 4B). These results suggest that the neutralizing antibodies may contribute to reduce viral load in this population.

Notably, in patients with 3S-specific antibodies, neutralizing titers were negatively correlated with viral load (Spearman *r* = − 0.6447, *P* = 0.0128) and proviral load (Spearman *r* = − 0.4567, *P* = 0.0217) (Fig. 4C). However, there was no correlation between neutralizing titers and viral load (Spearman *r* = -0.5357, *P* < 0.05) or proviral load (Spearman *r* = 0.2908, *P* = 0.1893) in patients with EC26-specific antibodies (Fig. 4D). These results suggest that neutralizing antibodies targeting the 3S epitope may directly contribute to reduce viral burden in the peripheral blood of these patients.

In a random subset of patients (N = 30) neutralization capacity was further assessed against a panel of tier 2 HIV-1 pseudoviruses representing different clades: TRO11 (subtype B), 246F3 (AC recombinant), CH119 (CRF07.BC), CE1176 (subtype C), BJOX2000 (CRF07.BC) and CNE55 (CRF01.AE). Consistent with the clade distribution in Portugal (Figure S1), the easiest virus to neutralize was subtype B TRO11 (neutralized > 30% by 23 samples, mean neutralization 55.4%) followed by BC recombinant CH119 (neutralized by 22 samples, mean neutralization 54.0%), BC recombinant BJOX2000 (20 samples, 53.3%), AC recombinant 246F3 (18 samples, 51.2%), subtype C CE1176 (18 samples, 50.0%) and AE recombinant CNE55 (13 samples, 39.4%) (Table 2).

**Table 2:**
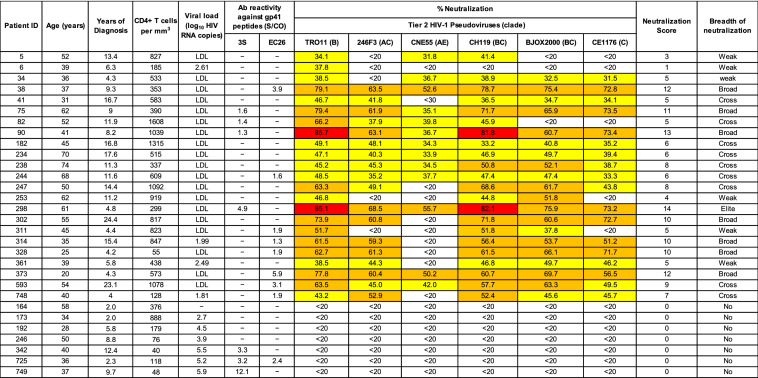
Neutralizing activity against tier 2 viruses.

(-) Absence of peptide-specific binding antibodies. S/CO- signal to cut-off optical density. LDL- lower than the detection level of 20 copies per ml of plasma. Percent neutralization was determined in TZM-bl cells with plasma samples diluted 1:100. White cells indicates < 20% of neutralization; Yellow cells indicate 20 to < 50% neutralization; light brown highlighting indicates 50% to < 80% neutralization; red highlighting indicates ≥ 80% neutralization.

**Figure 5:**
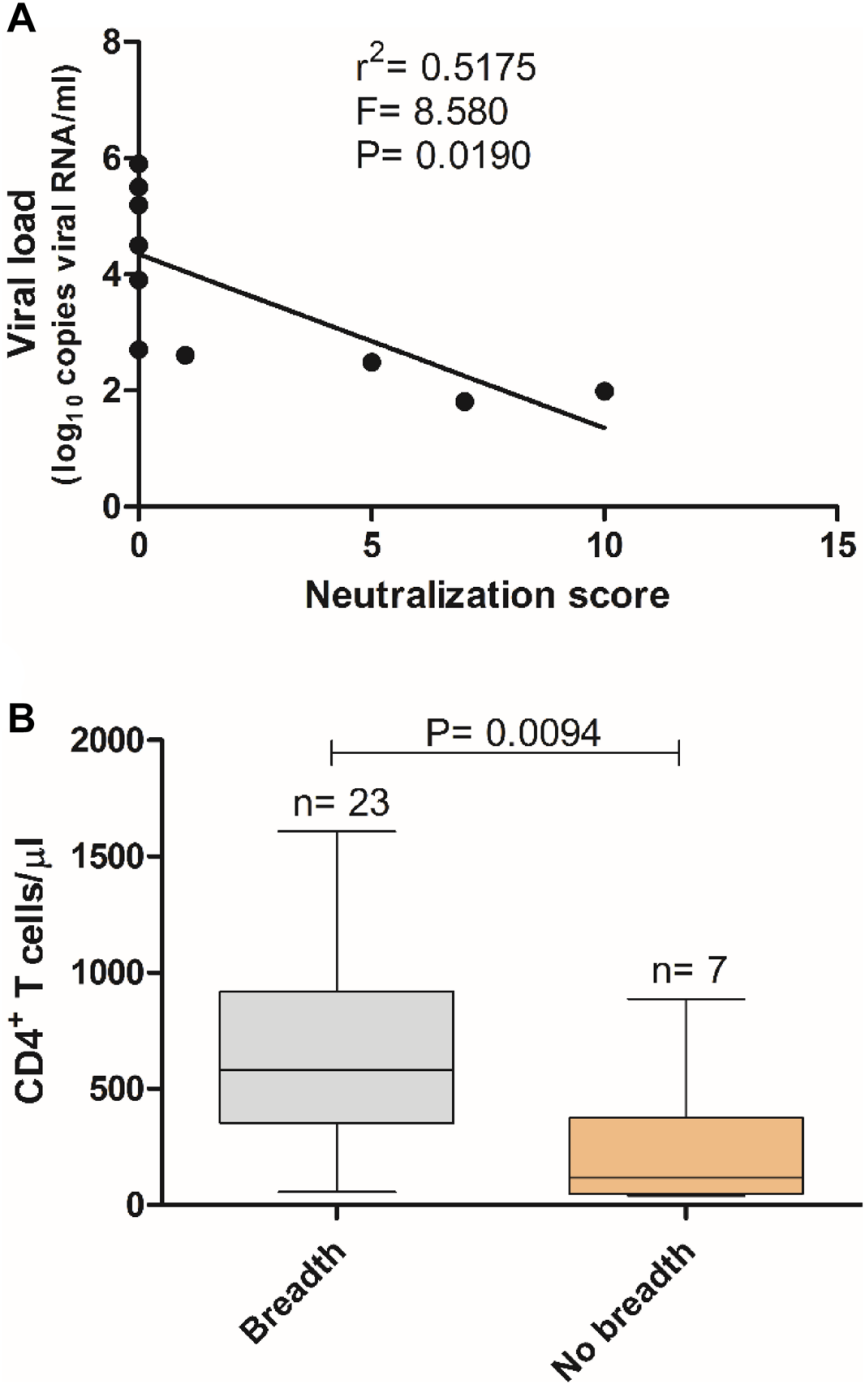
Viral load and CD4^+^ T cell counts in patients with or without broadly neutralizing antibodies as determined by neutralizing score. (**A**) Regression analysis; (**B**) Box and whiskers plot. Statistical significance was determined using the Mann–Whitney test. Lines denote median, maximum and minimum.

Neutralization breadth and potency was assessed using a score system that is based on the percentage of inhibition of each virus of the panel using a single plasma dilution^29^. Median neutralization score was 5.5, ranging from 0 to 14 (Table 2). The neutralizing profile of our population was as follows: elite neutralizers 3.3% (N = 1), cross neutralizers 30% (N = 7), broad neutralizers 23.3% (N = 11), weak 20% (N = 6) and no neutralizers 23.3% (N = 7). Neutralization scores were inversely correlated to viral load (Spearman *r* = − 0.8740, *P* = 0.0009), and patients that developed neutralization breadth had 4.9- fold higher number of CD4^+^ T cells relative to those that did not [median CD4^+^ T cell counts/ µl) (IQR) = 583 (353–919) vs. 118 (48–376), *P* = 0.0094] (Fig. 5). These results suggest that elicitation of broadly neutralizing antibodies directly contributes to decrease viral replication and prevent the decline in the number of CD4^+^ T lymphocytes.

**Figure 6:**
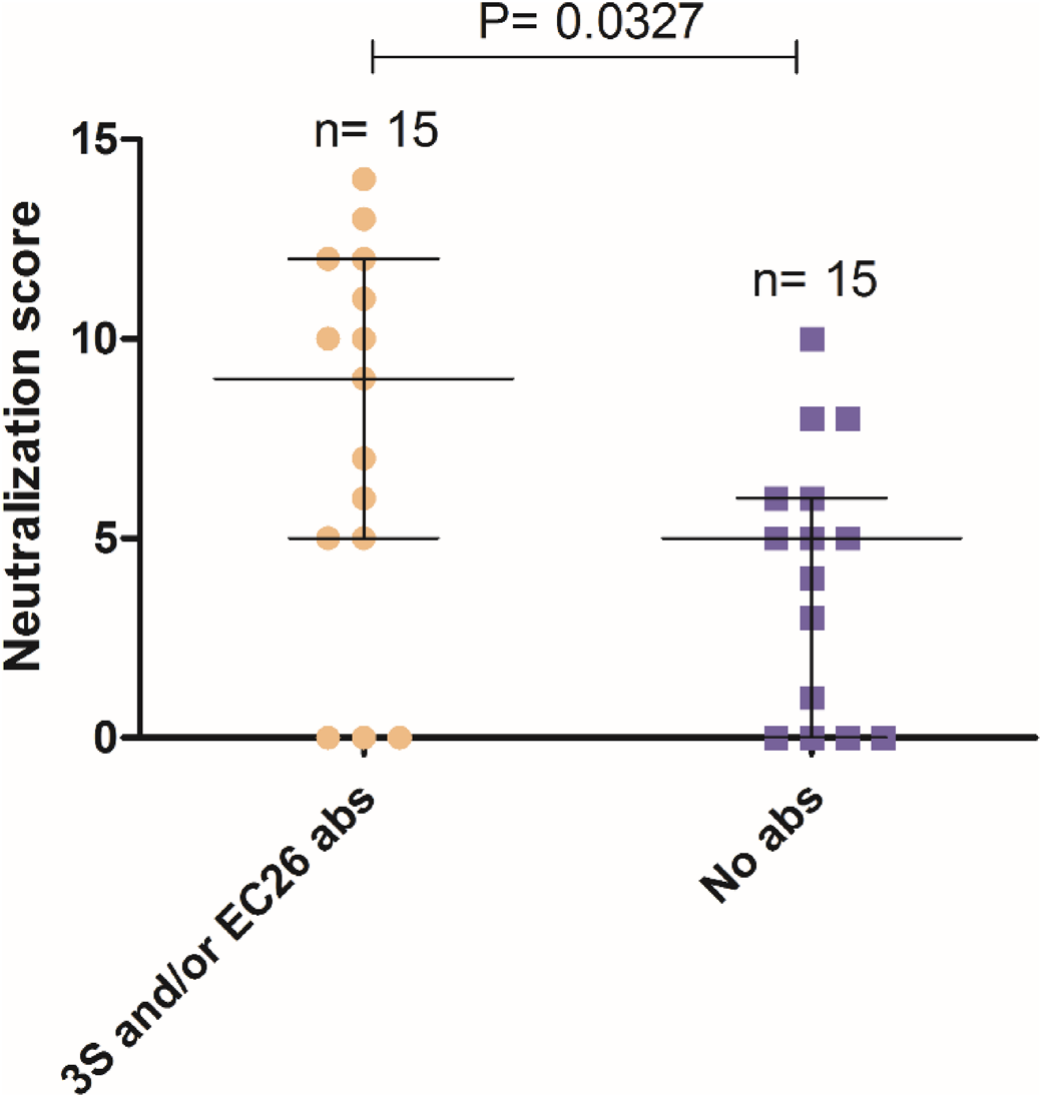
Neutralizing scores in patients with and without 3S or EC26 antibodies. Lines denote median and interquartile range. Statistical significance was determined using the Mann–Whitney test.

Noteworthy, patients with 3S and/or EC26-specific antibodies showed a 1.8 -fold higher median neutralization score than those without peptide antibodies [median score (IQR) = 9 (5–12) vs. 5 (0–6), *P* = 0.0327] (Fig. 6). These results suggest that 3S and/or EC26-specific antibodies contribute to the neutralization breadth and potency in HIV-1 infected patients.

## Discussion

**Figure 7:**
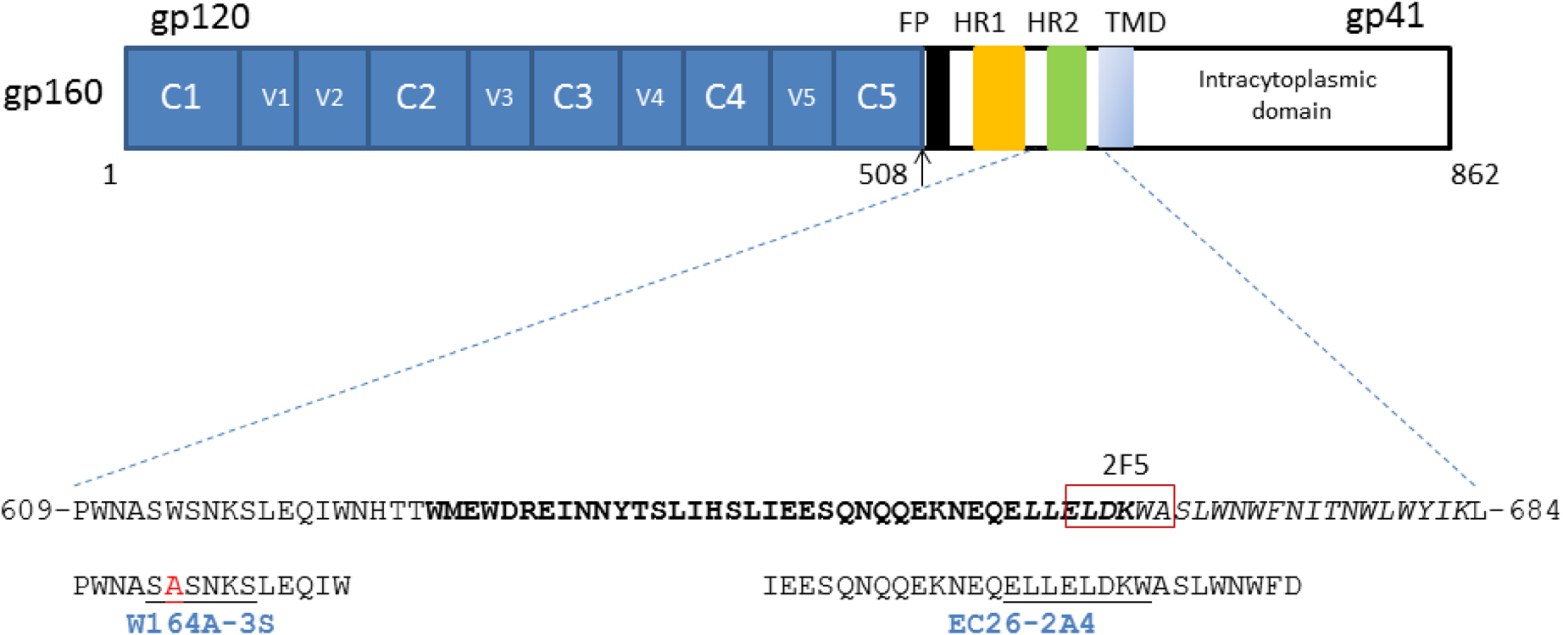
Sequence and location of W164A-3S and EC26-2A4 peptides in the transmembrane glycoprotein of HIV-1 (gp41, HXB2 reference strain numbering, GenBank: AF033819.3). Epitope sequences are indicated in underlined letters^18, 22, 52^. The W614A mutation in peptide W164A-3S is shown in red. The amino acid sequence of the heptad repeat 2 (HR2) region is indicated in bold letters (aa 628–665). TMD- transmembrane domain; FP- fusion peptide; HR1- heptad repeat 1. The membrane proximal external region (MPER) is shown in italic letters (aa 660–683)^6, 16^. The epitope of the broadly neutralizing antibody 2F5 is boxed^17^. In gp120, V1-V5 are hipervariable regions and C1-C5 are conserved regions.

We performed the first detailed characterization of the antibody response of HIV-1-infected patients against two peptides derived from the gp41 ectodomain region named 3S and EC26^18, 19, 22^. The 3S peptide used in this work is the W164A-3S modified version of the wild-type peptide and is located between the heptad repeat (HR)-1 and 2 regions immediately adjacent to the gp41 immunodominant region (aa 601–609)^30^. EC26 is located in the HR-2 region and its COOH-terminal part overlaps the NH2-terminal part of the MPER region and includes the epitope of the 2F5 broadly neutralizing antibody (662-ELDKWA-667) (Fig. 7)^17^. The antigenicity of 3S or EC26 has been previously studied in French and German HIV-1 infected individuals and their immunogenicity has been tested in mice, nonhuman primates and humans^18, 19, 23–26, 31–33^. Based on these results EC26 and, in particular, 3S are considered promising potential vaccine candidates^19, 24^. However, these gp41 regions vary significantly between and within HIV-1 clades which may affect their antigenicity and immunogenicity^28, 34^. Therefore, it is important to characterize the antigenicity of these peptides in other HIV-1 epidemics in other countries.

The antigenicity of 3S and EC26 was characterized in a large panel of HIV-1 infected individuals from Portugal. In contrast to France and Germany where > 75% of all patients are infected with subtype B, the HIV-1 epidemic in Portugal is more heterogeneous and is caused by subtypes G (38.6%) and B (35.3%), followed by 02_AG (5.5%), C (4.6%) and 14_BG (3.3%)^35–43^. Main secondary clades circulating in Germany and France are, respectively, C (4.2%) and 02_AG (6.5%) (Figure S1). We found that 9.8% of our patients had antibodies reacting against EC26 which is similar to German patients (8.5%) and 2.7-fold lower than French patients (26.8%)^20^. The most relevant amino acid residues for EC26 binding to plasma antibodies are L660, L663, D664, and K665^20^ but amino acids at positions 659, 667, 668 and 669 are also important for antibody binding and for HIV-1 neutralization^28, 34, 44^. L660, L663, D664 and L669 are conserved in all clades consistent with their role in determining the structure of gp41^16^. In contrast, amino acids at positions 659 and 665 are highly polymorphic in clades C, G and 02_AG, and amino acids at position 667 and 668 are highly polymorphic in clade C and highly conserved in the other clades (Figure S3)^28^. Therefore, the relatively low antigenicity of EC26 in Germany and Portugal may be related with the polymorphic nature at amino acids 659, 665, 667 and/or 668 in clade C and G isolates circulating in these countries. Consistent with this, earlier studies have shown that binding of monoclonal antibodies to this gp41 region is clade dependent^34^. Along the same lines, patients infected with clade C rarely produce antibodies binding to the 2F5 epitope^45^, and clade C resistance to 2F5 neutralization is due to clade C not having otherwise conserved K665 and A667^28, 46, 47^.

Comparing the antigenicity of EC26 with adjacent and overlapping antigenic regions, the frequency of patients with EC26 antibodies was 6.1 to 9.1-fold lower relative to Portuguese patients with antibodies against other HR2 peptides such as T20 (which contains the 2F5 epitope) and P3 (similar to T20 but lacking the 2F5 epitope)^11^, and 3.0 to 6.7-fold lower compared to patients from Spain^48^ and South Africa^45^ with antibodies against MPER peptides. Overall, these results are consistent with EC26 defining a new antigenic region in gp41^18^. The lower frequency of EC26-positive individuals relative to individuals with MPER antibodies suggest that EC26 is less exposed than the MPER region during infection^49^. Alternatively, P3 and T20 antibodies produced during infection, along with MPER antibodies, could mask or down-modulate B-cell responses to EC26^34, 50^. Nonetheless, as found for some MPER antibodies^51^, the presence of EC26 antibodies was associated with low viral load and neutralization breadth (see below) suggesting a potential role for these antibodies in controlling viral replication^18^.

Twice as many of our patients had antibodies reacting with 3S relative to French patients on ART (9.5% vs. 4.7%)^52^. With the exception of amino acid at position 618 which is S in all clades and T in 02_AG, the 3S epitope (positions 613–618) is highly conserved (Figure S3)^22^. However, position 619 is highly polymorphic in clade B and positions 620 and 621 are highly polymorphic in clades G and 02_AG. This variability likely explains the differences in 3S antigenicity in France and Portugal^28^. Despite the similar number of patients with antibodies reacting with 3S and EC26, the level of reactivity of 3S antibodies was significantly higher than EC26. Moreover, reactivity against 3S was unrelated with disease stage, as assessed by CD4^+^ T cell counts, but directly related with viral load in plasma since more viraemic than aviraemic patients produced 3S antibodies, and 3S antibody reactivity levels were higher in viraemic patients. This contrasts with an earlier study in France where 3S antibodies were detected exclusively in patients with high CD4^+^ T cell counts and undetectable viral load^52^.

Patients with antibody responses against both peptides were rare in our cohort (1.2%) indicating that the simultaneous exposure of the 3S and EC26 antigenic epitopes in gp41 during infection is uncommon. Double reactivity seemed to be independent of infection time, and all but one patient with double peptide reactivity had low to undetectable viral load, high CD4^+^ T cell counts, and exhibited potent (≥ 83% inhibition) tier 1 neutralization, suggesting that double reactivity to these gp41 regions might be important for virus control^21, 49^. Interestingly, in these patients the reactivity level against EC26 was similar to 3S and exceeded that observed in patients with single reactivity. This is consistent with a role for EC26 antibodies in the control of HIV replication^18^, and suggests that the viruses infecting these patients have an unusual gp41 conformation that enables an increased exposure of EC26 to the B cells. Finally, the finding that 3S and/or EC26 antibody reactivity levels may be high even after several decades of infection and ART is indicative of a continuous low level exposure of these antigenic regions to the immune system which increases the likelihood of development of broadly neutralizing antibodies^29, 53^.

We performed the first evaluation of neutralizing antibody response against HIV-1 in Portuguese patients. The majority (89.9%) of the patients developed tier 1 neutralizing antibodies and this was associated with undetectable viral load or significantly lower (1.8-fold lower) viral load levels relative to patients without neutralizing antibodies. Moreover, in the subset of patients that were tested for neutralization of a panel of tier 2 isolates from different clades it was found that breadth of neutralization was inversely correlated with plasma viral load and directly correlated with CD4^+^ T cell counts. These results are consistent with a role for broadly neutralizing antibodies in controlling viral replication and preventing the decline of CD4^+^ T lymphocytes^5, 26^.

We note that the neutralizing profile of our selected population was unusual in that most patients (56.7%) showed elite, broad or cross neutralization. This frequency of patients with bnAbs response is much higher than that reported for HIV-1-infected individuals in different geographies^29, 54–56^. For example, Rusert et al.^29^ in Switzerland found that most patients (79.1%) showed weak or no neutralization breadth and only 1.3% were elite neutralizers which compares to 3.3% in our cohort. This divergence is likely related with the small number of patients that we tested, and the different panel of tier 2 isolates used in the different studies. Time of infection of the patients and viral diversity has been shown to influence the developing of neutralization breadth^5, 28, 29, 53^. In this sense, the unusual set of HIV-1 clades found in Portugal along with the long infection time in most of our patients may have contributed to the high rate of broad neutralizers found in this study.

Despite the observed tendency for lower viral load in patients with binding EC26 antibodies, there was no correlation between EC26 antibodies and virus neutralization. However, in patients with anti-3S antibodies, significant negative correlations were observed between tier 1 neutralizing titers, proviral DNA levels and viral RNA levels. In addition, patients with 3S and/or EC26-specific antibodies showed a 1.8-fold higher median tier 2 neutralization score than those without peptide antibodies suggesting that, like MPER-specific antibodies^45, 53, 57^, 3S and EC26- antibodies contribute to the neutralization breadth and potency in HIV-1 infected patients. These results confirm and extend earlier findings indicating that 3S and EC26 are exposed to B cells during infection eliciting the generation of broadly neutralizing antibodies that contribute to reduce viral burden in the peripheral blood and prevent the decline in CD4^+^ T lymphocytes^18, 24, 26, 52^.

In conclusion, S3 and EC26 epitopes are exposed to B cells during HIV-1 infection in some patients and elicit the production of binding and broadly neutralizing antibodies that contribute to reduce viral burden. Our results provide further support for inclusion of both epitopes in HIV-1 vaccine candidates.

## Methods

### Study participants and ethics statement

The 695 patients participating in this study were HIV-1 positive and undergoing ART and they attended two central hospitals in Lisbon, Hospital de Santa Maria and Hospital Egas Moniz. Peripheral blood mononuclear cells (PBMCs) and plasma were isolated and cryopreserved from blood samples collected at a single time point (> 6 months after diagnosis). As negative controls, we used seronegative plasma samples from 20 healthy individuals. Ethics Committee of each hospital approved the study (DIRCLN-11ABR2014-141 and CECHLO-29JAN2014) and all patients provided written informed consent according to the declaration of Helsinki.

### Viruses, peptides and cells

HIV-1 pseudoviruses panel used in neutralization studies were produced as described recently^58^. W164A-3S and biotinylated EC26-2A4 peptides (designated as 3S and EC26, respectively) were provided by Vincent Vieillard of Centre de Immunologie et des Maladies Infectieuses (CIMI-Paris), France, and Ursula Dietrich of Institute Georg-Speyer-Haus, Germany. 8E5/LAV (Cat. Number 95) and TZM-bl (Cat. Number 8129) cells were obtained from the NIH AIDS Reagent Program. The sequence and location of the 3S and EC26 peptides are shown in Fig. 7.

### HIV-1 proviral DNA quantification in PBMCs and plasma viral load

Cellular DNA was extracted from PBMCs of HIV-1-infected patients with the QIamp DNA blood Mini kit protocol (Qiagen, USA). A quantitative real-time PCR assay based on gag gene primers and probes was used for proviral HIV-1 DNA quantification as described elsewhere^59^. The human hTERT gene was amplified as a positive internal control. Quantification was performed by optimizing a standard curve with serial dilutions of chromosomal DNA extracted from 8E5/LAV cells, which contain a single integrated copy of proviral DNA per cell, from 5 million HIV proviral DNA copies down to 5 copies. Limit of detection of the test is 5 copies and the range of the assay is 1 × 10^6^ copies. The quantification of HIV-1 viral load in the plasma was determined with the Roche AMPLICOR HIV-1 MONITOR test (limit of detection of 20 RNA copies/ml of plasma).

### Binding antibody response to 3S and EC26 peptides

The binding antibody response to 3S and EC26 peptides was assessed using an Enzyme linked immunosorbent assay (ELISA) test as previously described^19, 23^. Briefly, microtiter plates (MICROLON High Binding; Greiner, Merck, Germany) wells were coated with 3S (100 ng/well) and biotinylated EC26 (10 ng/well) peptides diluted in phosphate-buffered saline (PBS). For EC26, plates were first coated with streptavidin (200 ng/well) and then blocked with 2% BSA in PBS plus 0.05% Tween-20 (BPBS-T). After peptide coating and washing, the plates were blocked with PBS-T containing 5% skim milk powder (MPBS-T) for 1 h at room temperature (RT). Plasmas were diluted 1:100 in MPBS-T and added to the ELISA plate wells. After 1 h incubation at RT and washing, the wells were incubated 45 min at RT with HPR-conjugated Donkey anti-human IgG diluted 1:5000 in PBST (Jackson Immune Research). Colorimetric reaction was developed using 50 µl/well of KPL SureBlue TMB Microwell Peroxidase Substrate (sera care, Milford, MA, USA) and the reaction was stopped by adding 50 µl/well 1 N sulfuric acid. The optical density (OD) was read at 450 nm. The clinical cut-off (CO) value of the assays was determined using samples from healthy HIV-seronegative subjects (N = 10) and calculated as the mean optical density (OD) value of HIV-seronegative samples plus 3 times the standard deviation. The results of the assay were expressed quantitatively as OD clinical sample (S) / OD cut-off [S/CO] ratios. Samples with ratio > 1 were considered seroreactive.

### Neutralization assays

The presence of neutralizing antibodies in plasma samples was assessed using a tier 1a (easy to neutralize) isolate (HIV-1 NL4.3) and a panel of 6 tier 2 (difficult to neutralize) reference pseudoviruses and a luciferase-based assay in TZM-bl cells as previously described^60^. This assay measures a decrease in luciferase expression following a single round of viral infection. For neutralization assays heat-inactivated (56 °C/1 h) plasmas were twofold serially diluted (starting at a 1:100), incubated for 1 h at 37ºC with each pseudovirus [TCID of 50,000–75,000 relative luminescence units (RLU) equivalents] and then added to TZM-bl cells (1 × 10^4^ cells/well) in 10% D-MEM medium containing DEAE-Dextran (Sigma-Aldrich, USA) at a final concentration of 18 μg/ml. After 48 h, 150 µl of cell supernatant was removed and 100 µl per well of ONE-Glo luciferase assay substrate reagent (Promega, Madison, Wisconsin, USA) was added and luminescence was read on a luminometer (TECAN Infinite 200 Microplate Reader, Tecan Trading AG, Switzerland). Antibody neutralization values represent the percentage of inhibition of viral infection (measured by luciferase activity) at each plasma dilution relative to a pool of seronegative plasma samples (control) and were calculated as follows: percentage viral inhibition = [1 − (luciferase RLUs with positive plasma samples/luciferase RLUs negative plasma samples)] × 100. The 50% end-point dilution (ED50) was defined as the reciprocal plasma dilution that cause 50% reduction in RLU compared with virus control.

To evaluate the neutralizing potency and breadth against tier 2 isolates we adapted a recently developed scoring system^29^. A score of 0 was attributed when inhibition percentage of a given virus was < 20%, a score of 1 when inhibition ranged between 20% and < 50%, a score of 2 for 50% to < 80% inhibition and a score of 3 for ≥ 80% inhibitions. The overall neutralization score was obtained by adding the scores against the 6 virus of the tier 2 panel and reflects neutralization potency and breadth for any given plasma. Plasma samples with scores 14–18 were classified as elite neutralizers, 10–13 as broad neutralizers, 5–9 as cross neutralizers, 1–5 as weak neutralizers and 0 as no neutralizers.

### Statistical analysis

The statistical analysis was performed with GraphPad Prism version 5.01 (GraphPad Software Incorporated, San Diego, California, USA). The Mann–Whitney test and Fisher’s exact test were used to compare differences between groups. The Spearman rank test was used to quantify the magnitude and direction of the correlation between antibody neutralization titers and viral and proviral load. All hypothesis tests were two-tailed and P values < 0.05 were considered significant.

## Supplementary Information

Supplementary Information
