# Supplementary material for: Antibody response against selected epitopes in the HIV-1 envelope gp41 ectodomain contributes to reduce viral burden in HIV-1 infected patients
Source: Sci Rep. 2021 Apr 26;11:8993. doi: 10.1038/s41598-021-88274-9 (PMC8076315; doi:10.1038/s41598-021-88274-9)
Supplement: Supplementary file 1 — Supplementary Information [file 41598_2021_88274_MOESM1_ESM.docx]

**Title: Antibody response against selected epitopes in the HIV-1 envelope gp41 ectodomain contributes to reduce viral burden in HIV-1 infected patients**

Rute Marcelino ^1,5,6^, Filipa Gramacho ^2^, Francisco Martin ^5^, Pedro Brogueira ^4^, Nuno Janeiro ^2,3^, Claudia Afonso ^2,3^, Robert Badura ^2,3^, Emília Valadas ^2,3^, Kamal Mansinho ^4^, Luís Caldeira ^2,3^ Nuno Taveira ^5,6^ and José M. Marcelino ^1,5,6^.

^1^ Global Health and Tropical Medicine-GHTM, Instituto de Higiene e Medicina Tropical-IHMT, Universidade Nova de Lisboa-UNL, Lisboa, 1349-008 Lisboa, Portugal.

^2^  Hospital de Santa Maria-HSM, Centro Hospitalar Lisboa Norte-CHLN, E.P.E., Lisboa, 1649-028 Lisboa, Portugal.

^3^ Clínica Universitária de Doenças Infeciosas, Faculdade de Medicina, Universidade de Lisboa-UL, Lisboa, 1649-028 Lisboa, Portugal.

^4^  Serviço de Doenças Infeciosas, Hospital Egas Moniz-HEM, Centro Hospitalar Lisboa Ocidental-CHLO, E.P.E., Lisboa, 1349-019 Lisboa, Portugal.

^5^ Instituto de Investigação do Medicamento (iMed.ULisboa), Faculdade de Farmácia, Universidade de Lisboa, Lisboa, 1649-003 Lisboa, Portugal.

^6^ Centro de Investigação Interdisciplinar Egas Moniz (CiiEM), Instituto Universitário Egas Moniz, Monte de Caparica, 2829-511 Monte de Caparica, Portugal.

* corresponding. josemarcelino@ff.ulisboa.pt

**Supplementary information**

**Diversity of the gp41 region comprising the 3S and EC26 peptides in different HIV-1 clades.** Amino acid sequences comprising the 3S and EC26 peptides and the MPER region (positions 609-683) from the dominant HIV-1 clades in Portugal, Germany and France, i.e. B, C, G and 02_AG, were retrieved from the HIV sequence database at Los Alamos, US (<http://www.hiv.lanl.gov/>) (**Figure S1**). This amounted to 2731 B sequences, 936 C sequences, 138 02_AG sequences and 65 G sequences. Sequences were manually curated to eliminate those with low quality and then aligned using MAFFT (<https://mafft.cbrc.jp/alignment/server/>). The diversity at each amino acid position in the alignment was measured with Shannon’s entropy using the dedicate tool available at the HIV sequence database at Los Alamos website. Median entropy values were significantly higher for clades B and C relative to clades G and 02_AG demonstrating that in this part of gp41, B and C clades are more diverse then clades G and 02_AG (**Figure S2**).


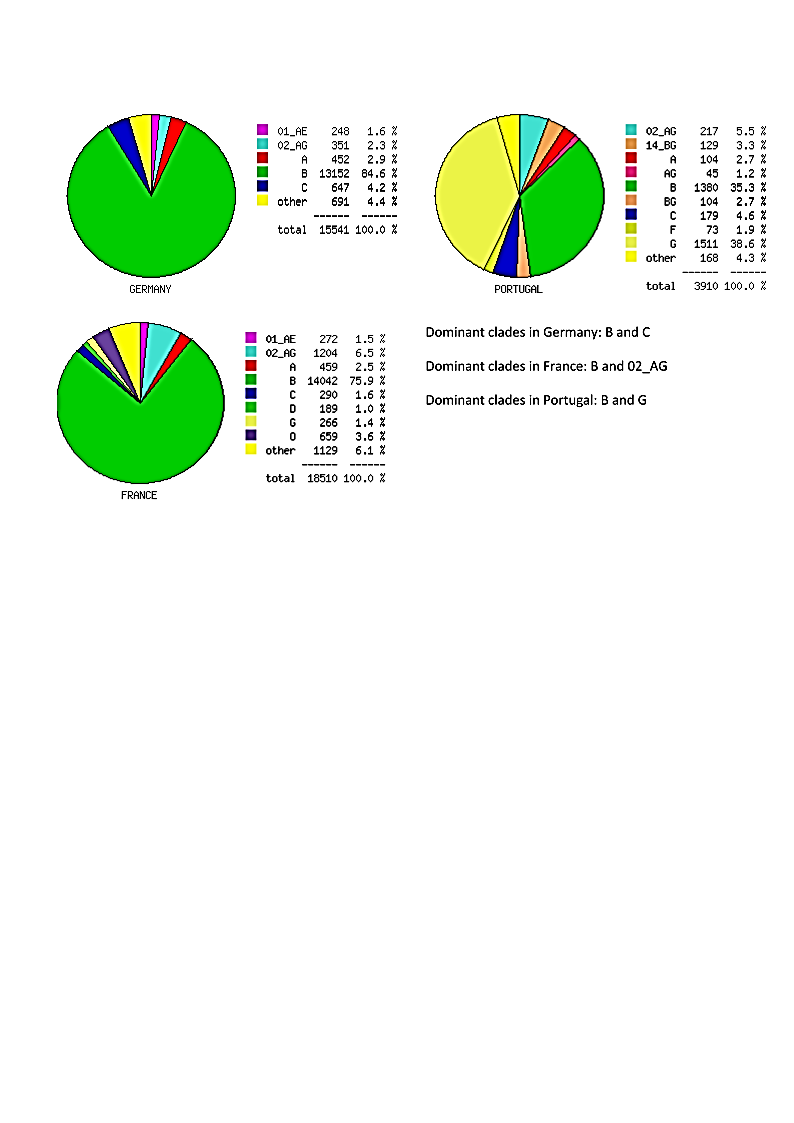


**Figure S1**. HIV-1 clade composition in Portugal, Germany and France in 2020. Plots were based on sequences deposited at the HIV sequence database at the Los Alamos website in September 06 2020.


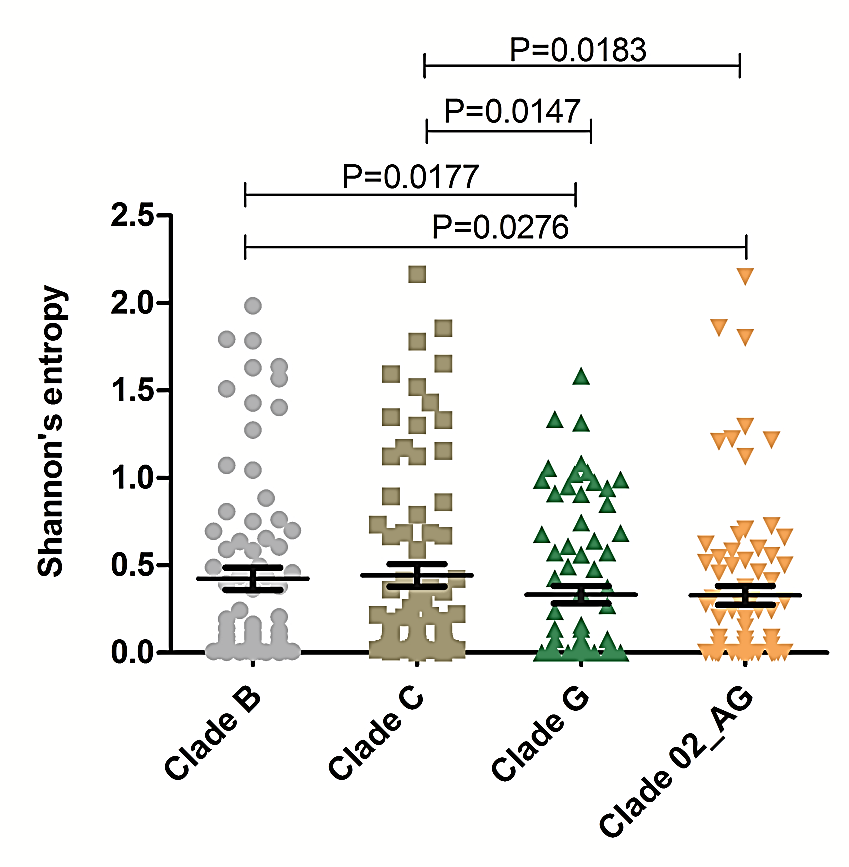


**Figure S2**. Diversity of the gp41 region comprising the 3S and EC26 peptides (aa 609-684) in HIV-1 clades B, C, G and 02_AG as assessed by Shannon’s entropy. These clades prevail in the HIV-1 epidemics in Portugal, Germany and France. P values were obtained using the Mann Whitney test.

To look more closely at the diversity in the 3S and EC26 peptide region, LOGO plots were done for each clade using the respective tool available at the HIV sequence database at Los Alamos, US (**Figure S3**). The results give a good perspective of the amino acid residues that are more variable in these regions in the four clades.


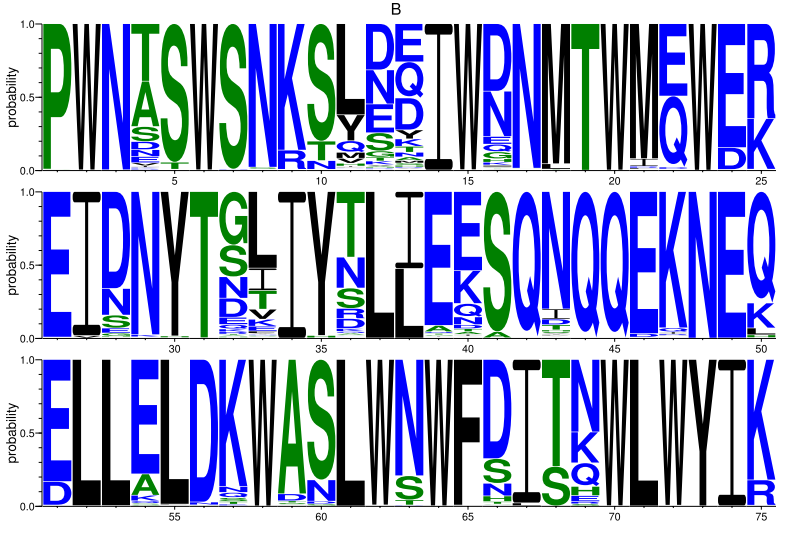


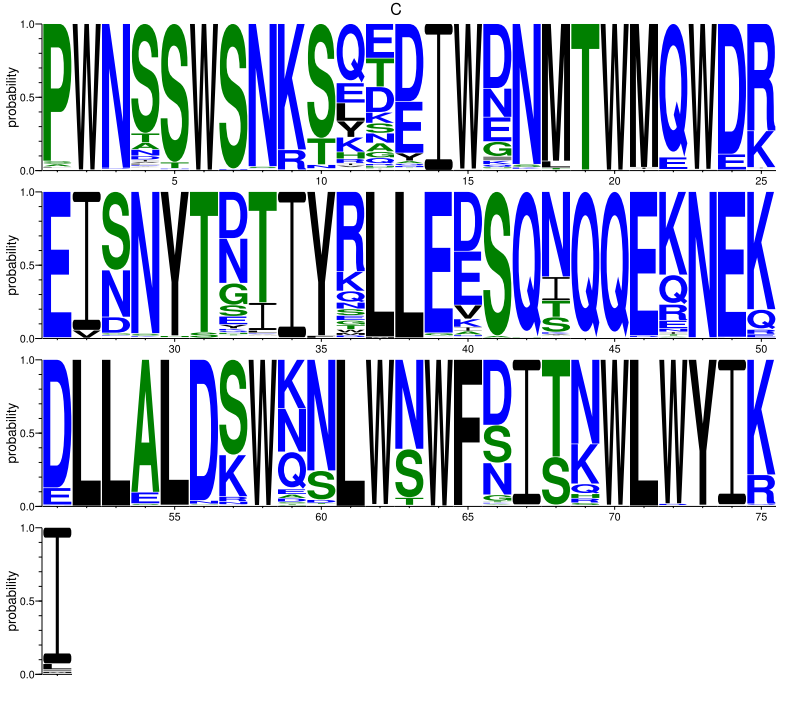


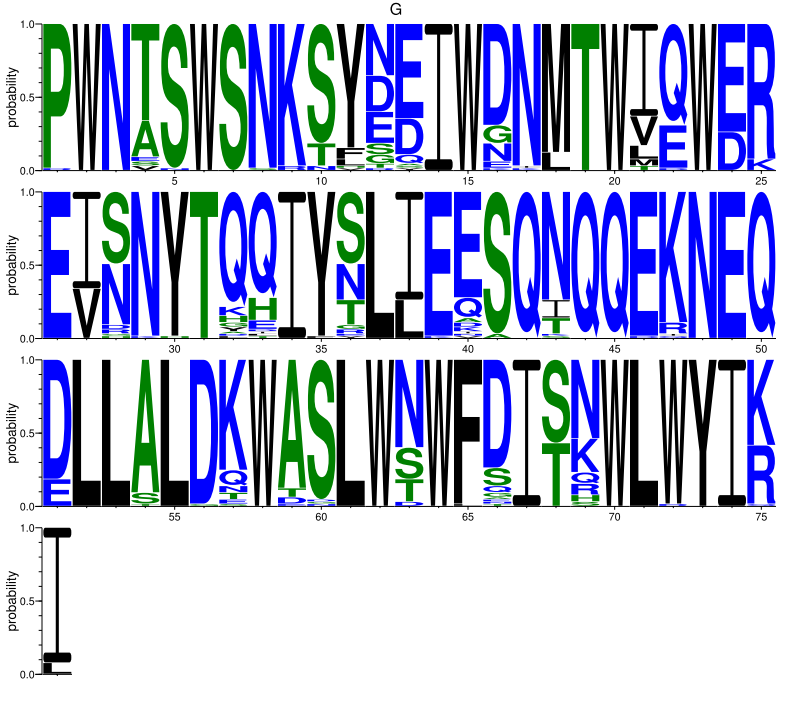


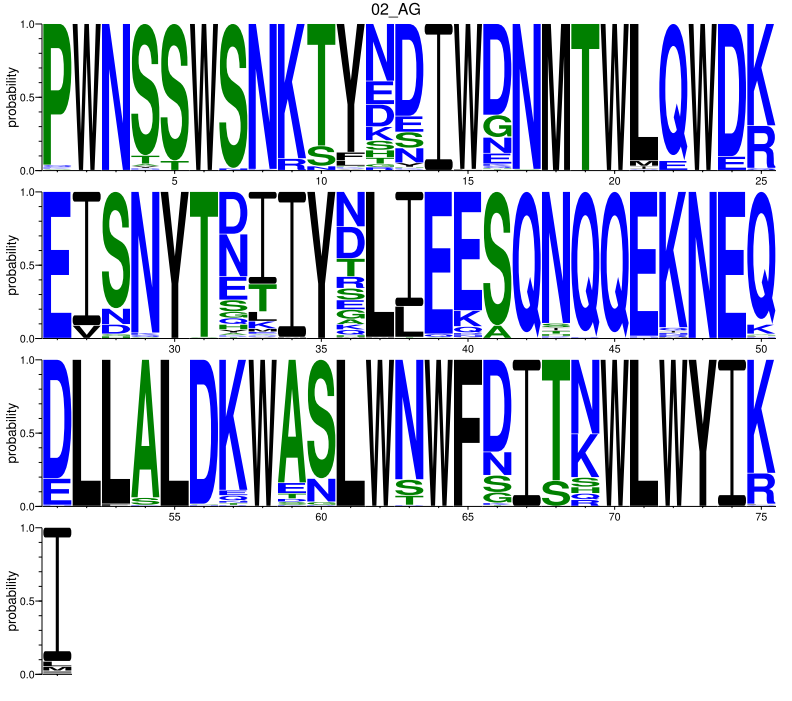


**Figure S3.** LOGO plots of the gp41 region comprising 3S and EC26 peptides (residues 606-683) for clades B, C, G and 02_AG.

**Peptide binding reactivity and infection length.** Binding reactivity against EC26 tended to increase with infection length and to decrease against 3S (**Figure S4**) but there was no significant correlation between binding and neutralizing antibody responses and infection length in this population (**Figure S5**).


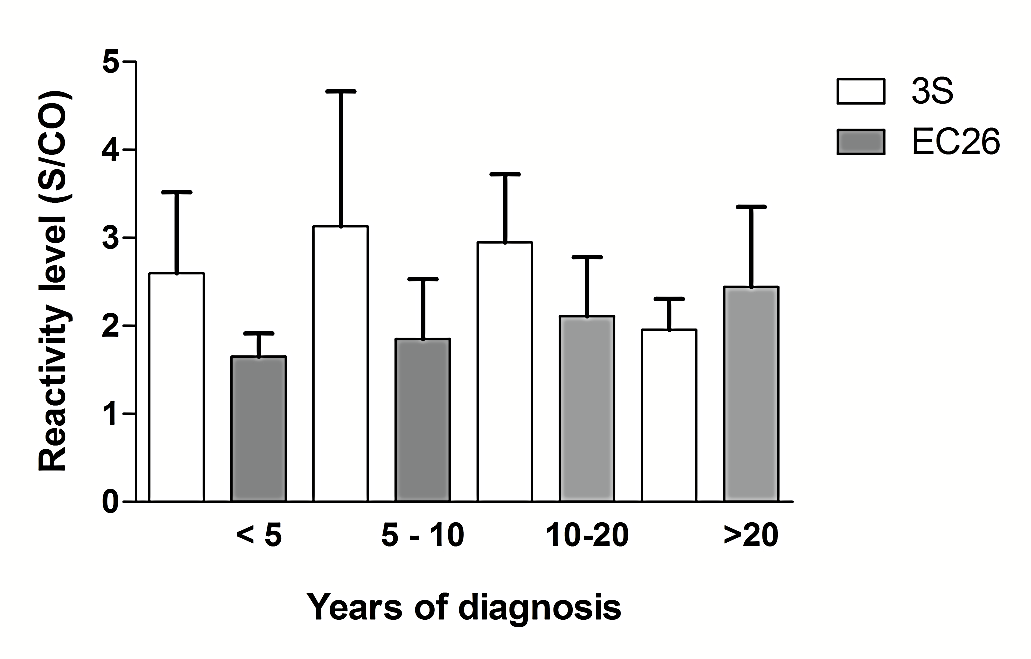


**Figure S4**. Binding antibody reactivity against 3S and EC26 peptides in individuals with different infection periods after diagnosis.

**
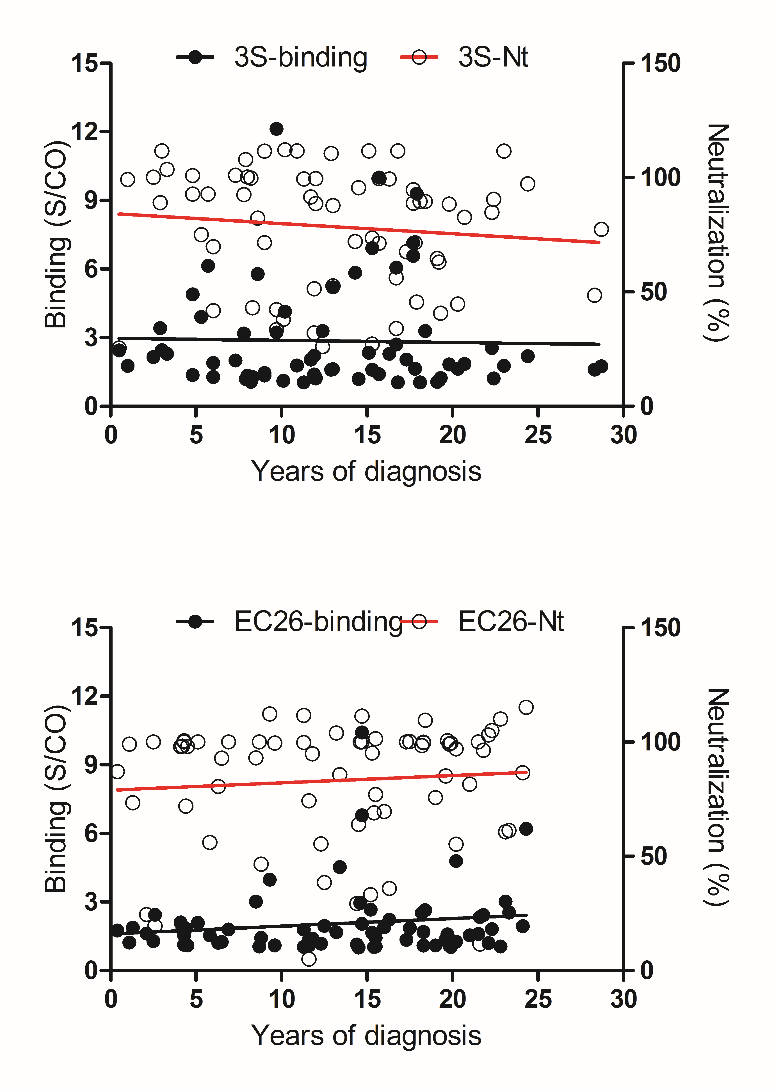
**

**Figure S5**. Binding antibody reactivity against 3S and EC26 peptides and antibody neutralization in individuals with different infection periods after diagnosis. Neutralization was tested against the tier 1 HIV-1 NL4.3 isolate. The linear regression line is shown.

**Table S1.** Characteristics of the patients with double reactivity against 3S and EC26 peptides
